# Evolving quality improvement support strategies to improve Plan–Do–Study–Act cycle fidelity: a retrospective mixed-methods study

**DOI:** 10.1136/bmjqs-2017-007605

**Published:** 2019-03-18

**Authors:** Chris McNicholas, Laura Lennox, Thomas Woodcock, Derek Bell, Julie E Reed

**Affiliations:** 1 NIHR CLAHRC NWL, Chelsea and Westminster Hosptial, Imperial College London, London, United Kingdom; 2 Improvement Team, Office of Medical Director, Imperial College Healthcare NHS Trust, London, United Kingdom

**Keywords:** quality improvement, PDSA, quality improvement methodologies, Plan-Do-Study-Act

## Abstract

**Background:**

Although widely recommended as an effective approach to quality improvement (QI), the Plan–Do–Study–Act (PDSA) cycle method can be challenging to use, and low fidelity of published accounts of the method has been reported. There is little evidence on the fidelity of PDSA cycles used by front-line teams, nor how to support and improve the method’s use. Data collected from 39 front-line improvement teams provided an opportunity to retrospectively investigate PDSA cycle use and how strategies were modified to help improve this over time.

**Methods:**

The fidelity of 421 PDSA cycles was reviewed using a predefined framework and statistical analysis examined whether fidelity changed over three annual rounds of projects. The experiences of project teams and QI support staff were investigated through document analysis and interviews.

**Results:**

Although modest, statistically significant improvements in PDSA fidelity occurred; however, overall fidelity remained low. Challenges to achieving greater fidelity reflected problems with understanding the PDSA methodology, intention to use and application in practice. These problems were exacerbated by assumptions made in the original QI training and support strategies: that PDSA was easy to understand; that teams would be motivated and willing to use PDSA; and that PDSA is easy to apply. QI strategies that evolved to overcome these challenges included project selection process, redesign of training, increased hands-on support and investment in training QI support staff.

**Conclusion:**

This study identifies support strategies that may help improve PDSA cycle fidelity. It provides an approach to assess minimum standards of fidelity which can be replicated elsewhere. The findings suggest achieving high PDSA fidelity requires a gradual and negotiated process to explore different perspectives and encourage new ways of working.

## Introduction

Quality improvement (QI) approaches continue to grow in popularity in healthcare. This increased emphasis and uptake of the approaches needs to be balanced by an understanding of how to ensure their effective use to enable the delivery of improvements in patient care. Without such assurances there is a danger that QI remains a ‘slogan of intent’ to improve quality rather than an authentic application of the concepts in practice.^1 2^

The Plan–Do–Study–Act (PDSA) cycle method is widely recommended as an effective approach to QI; however, previous research has demonstrated that the fidelity of the method reported in peer-reviewed literature is low^3^ and barriers are encountered in its use.^4–6^ PDSA cycle fidelity has been defined as the degree to which a PDSA cycle is carried out in accordance to the guiding principles of its use (table 1).^3^ Measuring fidelity of the PDSA cycles demonstrates whether the method has been used as intended, which in turn can inform assessments as to whether its desired benefits have been achieved: learning to inform the evolution of a change idea to support achievement of a stated aim.^7^ There is little overarching empirical evidence, however, of the fidelity of PDSA used by front-line teams or understanding of factors that may influence the fidelity of PDSA cycle use.^8^

**Table 1:** PDSA cycle fidelity assessment

PDSA cycle conduct principle	Fidelity assessment (yes/no)	PDSA cycles included in the analysis
Documentation	Were all cycle stages of the PDSA cycle documented?	All initiated PDSA cycles (PDSA cycles with a documented ‘Plan’).
	Was the ’Study’ stage documented in the past tense (indicating that the PDSA cycle was executed)?	All fully documented PDSA cycles.
Learning activity	Was the PDSA cycle used to structure a learning activity (cycle documenting a test of change or collection of information)?	All fully documented PDSA cycles.
Prediction	Was an explicit prediction documented?	All fully documented PDSA cycles describing a learning activity.
Iterative cycles	Was the PDSA cycle within an iterative series of PDSA cycles?	All fully documented PDSA cycles.
Incremental testing scale	Was the PDSA cycle within an iterative series of PDSA cycles increasing scale?	All fully documented PDSA cycles within an iterative series.
Use of data over time	Was the PDSA cycle within an iterative series of PDSA cycles using regular data over time?	All fully documented PDSA cycles within an iterative series.

PDSA, Plan-Do-Study-Act.

This study explores the PDSA cycle conduct of front-line healthcare improvement teams supported by the National Institute for Health Research (NIHR) Collaboration for Leadership in Applied Health Research and Care (CLAHRC) Northwest London (NWL) programme 2008–2013. It takes advantage of the documentation collated by the CLAHRC NWL programme to conduct a retrospective study. Specifically it aims to (1) assess the fidelity of a range of PDSA cycles documented in real time by front-line improvement teams; (2) determine whether any change in PDSA fidelity occurred over time; and (3) explore the strategies deployed by the programme team to support and improve the use of PDSA cycles.

By retrospectively capturing the experience of the programme and project teams, we aim to provide insight into the reality of using the PDSA cycle method and providing support to teams to do so. The overall intention of the paper is to support future programmes and project teams in using the method effectively to improve patient care.

## Methods

### Sample

Between 2009 and 2012 the NIHR CLAHRC NWL programme supported 39 projects.^9–12^ Using a QI collaborative structure, a central programme team provided training and support to help front-line improvement teams use a suite of QI methods, including PDSA cycles, to improve the quality of healthcare through the implementation of research evidence into practice. The QI support team were made up of members of the programme and were from a range of backgrounds—clinical, managerial, information analysts and researchers. Each project team was assigned a main point of contact in the QI support team. The majority of the QI support team stayed the same throughout the programme, with one senior member moving on and being replaced after 2 years, and four additional junior posts starting in 2010. The support provided by the programme is referred to as ‘QI Support Strategies’.

**Figure 1:**
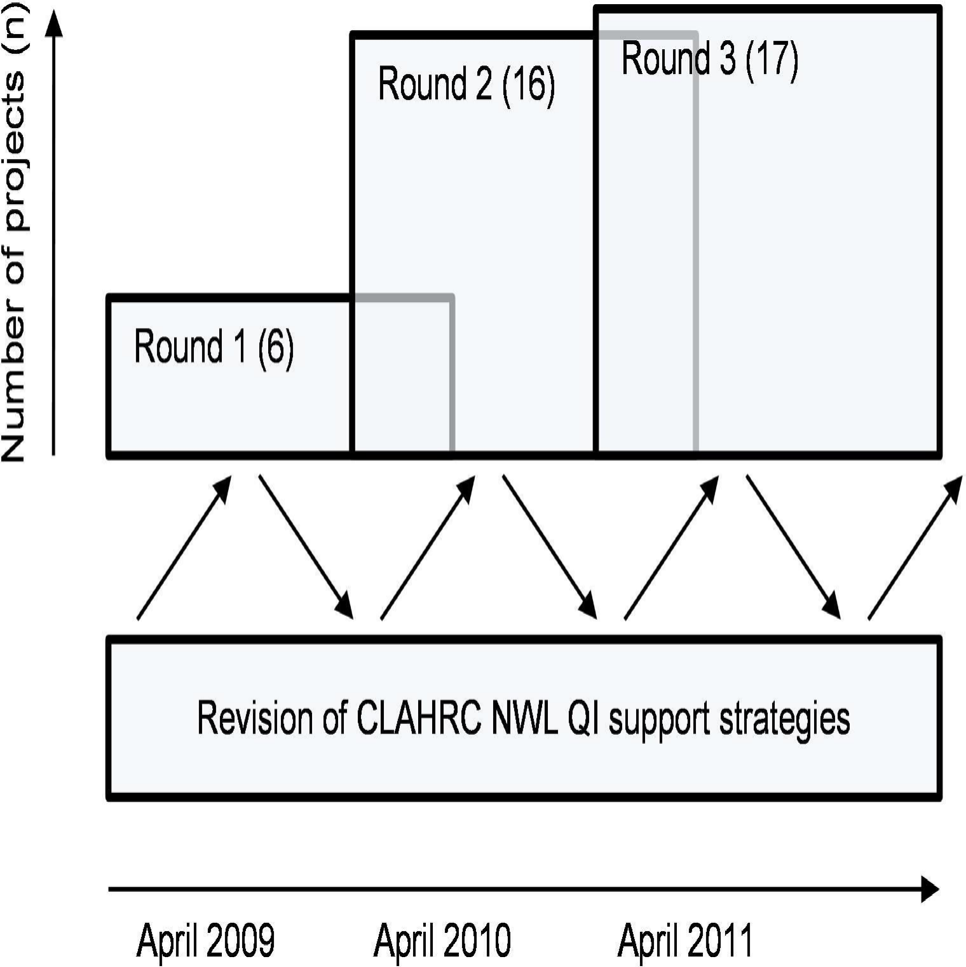
’Round’ project initiation approach of National Institute for Health Research Collaboration for Leadership in Applied Health Research and Care (CLAHRC) for Northwest London (NWL). QI, quality improvement.

The projects were conducted over the three rounds of projects, each round lasting 18 months, with 6 starting in April 2009, 16 in 2010 and 17 in 2011 (figure 1).^13^ Project team members tended to be QI novices, with little or no prior QI experience. No entire team participated in more than one round, although a small number of individuals participated in different project teams over more than one round. The initiation of projects annually was purposeful so that teams overlapped and shared experiences, and so that modifications to the QI support strategies could be made based on feedback of both the programme and project teams.

### Data collection

Each project documented their use of PDSA cycles in real time on an online tool, the Web Improvement Support for Healthcare system.^14^ A total of 421 PDSA cycles were documented and are included in the study.

Feedback from project teams about the use of PDSA cycles (and other QI methods) and QI support strategies was collated throughout the programme. This included project reports (n=96, including details of how QI methods were used); minutes from formal project review meetings (n=84, including discussion of project team perceptions of QI methods and QI support strategies, held at 6 monthly interviews); and teaching and support materials (including planning documents, slides and activity handouts). In total, 180 project documents were assessed (number of documents review per round: R1=11, R2=80, R3=89) and training materials from 20 events teaching or referring to PDSA cycles. In addition, to triangulate data derived from the document analysis, interviews were conducted retrospectively after all three rounds of projects had been completed. Three programme team members who had been involved in teaching and support of PDSA across all three rounds of the programme were interviewed. The data from the interviews were intended primarily to clarify and explain our findings.

### Analysis

#### What was the fidelity of conduct of all PDSA cycles against the core principles of the method?

A structured framework was used to assess the fidelity of PDSA cycle use against the key principles of the method^3^ (table 1). The documented PDSA cycles of the CLAHRC NWL project teams were assessed by deductive content analysis against this framework.^15 16^

Two reviewers (CM and LL) first coded a third of the 421 cycles against the principles in Microsoft Excel. Before coding, they were familiar with and had discussed the principles outlined in Taylor *et al*’s^3^ systematic review of PDSA cycles (of which CM was an author). They also reviewed a small number of PDSA cycles together to learn how to apply the framework. Both reviewers had completed QI training on a range of methods, including PDSA cycles. The reviewers were blinded from the project name and round, and while they may have delivered training for the teams they were not involved in the delivery of the projects. Intercoder reliability, as indicated by Cohen’s kappa, ranged between 1 and 0.77, with percentage agreement between 100% and 82%. Discrepancies were resolved by discussion and consensus and a shared understanding was developed. The remainder of the cycles were then coded by one reviewer (CM).

#### How did PDSA cycle fidelity change over time?

The quantitative outputs for the measures of fidelity from the first stage of analysis were divided by the year the project teams were initiated. A one-way analysis of variance and post-hoc t-tests were first used to determine change in the mean number of PDSA cycles conducted per project overtime. χ^2^ tests and a subsequent trend test, the Marascuilo procedure, were used to assess the significance in changes observed for each fidelity assessment over time (see online supplementary appendix 1 for further details).^17^

10.1136/bmjqs-2017-007605.supp1 Supplementary data

#### What QI support strategies were used by the programme and how did these change over time?

The experiences of project teams and QI support strategies used by the QI support team were explored through document and interview analysis.

Project reviews and training materials were initially reviewed to identify high-level themes relating to PDSA cycle conduct and QI support strategies. These themes informed the interview questions with QI support team members. Selected training materials were brought to interviews to act as prompts. Drawing on both documentation and interview transcripts, a full thematic inductive analysis using the constant comparative method was undertaken to identify themes relating to the QI support strategies used and the experiences of both the QI teams and QI support team. Detailed open-coding of themes within text were identified along with code definitions. These were grouped to high-level categories before further conceptualisation within each category. Coders met to discuss and refine coding in an iterative manner. All data (documentation and interview transcripts) were also coded to project round(s) that they related to. Themes were discussed with other authors and other members of the QI support team to support sense-making of the analysis in light of the historical development and conduct of the programme, and to discuss and clarify gaps in analysis. Full roles of the authors in the data analysis are presented in online supplementary appendix 1.

## Results

### What was the fidelity of conduct of all PDSA cycles against the core principles of the method?

**Figure 2:**
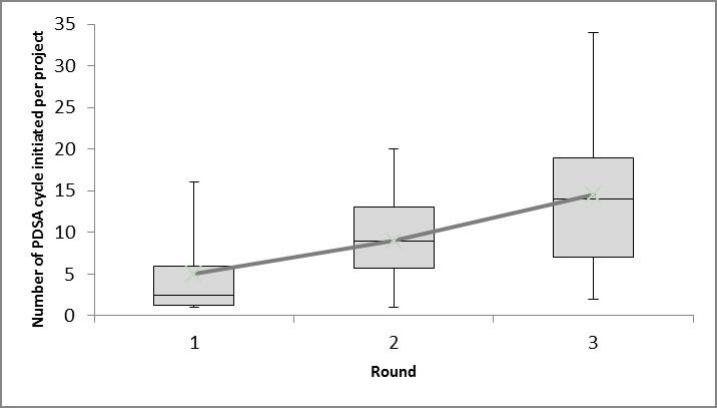
Box and whisker diagram of the number of Plan–Do–Study–Act (PDSA) cycles per project across the three rounds.

A total of 421 PDSA cycles were documented and included in the study. There was a statistically significant increase in the mean number of PDSA cycles initiated by project teams across the three rounds (figure 2) (p<0.05) (online supplementary appendix 1 provides further details on the analyses).

Over the period of study, 2% (7/421) of PDSA cycles reviewed adhered to all six measures of fidelity, 12% (49/421) adhered to >5 principles and 29% (121/421) adhered to >4 principles as described in the framework. Table 2 presents individual results by each measure of fidelity. Only PDSAs with full documentation were included in full fidelity analysis (299), the rest (122) being excluded from further analysis.

**Table 2:** Change in measures of PDSA cycle fidelity over round of project initiation

Principle	Measure		Round 1	Round 2	Round 3	All	P value
Documentation	All PDSA cycle stages documented	Cycles adhering to principle	15	93	191	299	<0.001
		Cycle sample	30	144	247	421	
		%	**50.0**	**64.6**	**77.3**	**71.0**	
	’Study’ section documented in past tense	Cycles adhering to principle	10	67	176	253	<0.001
		Cycle sample	15	93	191	299	
		%	**66.7**	**72.0**	**92.1**	**84.6**	
Learning activity	Learning activity present in PDSA cycle	Cycles adhering to principle	15	90	189	294	NA
		Cycle sample	15	93	191	299	
		%	**100**	**96.8**	**99.0**	**98.3**	
Prediction	Explicit prediction documented in PDSA cycle	Cycles adhering to principle	0	3	33	36	0.001
		Cycle sample	15	90	189	294	
		%	**0.0**	**3.3**	**17.5**	**12.2**	
Iterative cycles	PDSA cycle within iterative series of 2 or more cycles	Cycles adhering to principle	0	48	115	163	<0.001
		Cycle sample	15	93	191	299	
		%	**0.0**	**51.6**	**60.2**	**54.5**	
Small-scale testing	PDSA iterative series increasing testing scale	Iterative series adhering to principle	NA	3	16	19	0.113
		Cycle sample	NA	19	45	64	
		%	**NA**	**15.8**	**35.6**	**29.7**	
Use of data over time	PDSA iterative series using regular data over time	Iterative series adhering to principle	NA	7	22	29	0.376
		Cycle sample	NA	19	45	64	
		%	**NA**	**36.8**	**48.9**	**45.3**	

Bold values are calulated as the percentage of the cycle sample that adhere to the principle.

NA, not applicable; PDSA, Plan-Do-Study-Act.

### How did PDSA cycle fidelity change over time?

Improvements in fidelity were observed across project rounds for all PDSA cycle principles, except for the presence of a *learning* activity within PDSA cycles which was high (above 98% of cycles) across all three rounds (table 2). These improvements were statistically significant for *documentation* (all PDSA cycle stages documented, p<0.001, moderate improvement 50%–77%; ‘Study’ documented in past tense, p<0.001, moderate improvement 67%–92%), *predictions* (explicit prediction documented, p=0.001, modest improvement 0%–18%) and *iterative cycles* (PDSA cycle within iterative series of cycles, p<0.001, substantial improvement 0%–60%). Improvements were seen for *incremental scale* and *use of regular data over time*, but these findings were not statistically significant. The seven cycles adhering to all indicators of fidelity were all from final round projects. Online supplementary appendix 2 presents the full statistical results.

10.1136/bmjqs-2017-007605.supp2 Supplementary data

### What QI support strategies were used by the programme and what were their experiences of introducing PDSA to QI novice teams?

Overall, thematic analysis of all data identified three areas of challenge for project teams using PDSA: intention to use, understanding of how to use and the application in practice. They were evident through three corresponding assumptions, described by interviewees, in designing the original QI support strategies: a belief that people would be motivated and willing to use PDSA, that PDSA was easy to understand, and that PDSA was easy to apply in practice.

“Our assumption was that it was quite straightforward – you teach people and they use the method.” (QI support team member, interviewee 2)

By assuming that PDSA was easy and that project teams would be receptive to its use, the original QI support strategies failed to address the challenges encountered, particularly in the first round of the projects. The QI support strategies in round 1 (table 3) were felt to have exacerbated these issues and were seen as a contributing reason for low levels of understanding and intention resulting in the low levels of PDSA use and PDSA fidelity identified in the quantitative analysis for round 1.

**Table 3:** Original and revised QI support strategies and reported consequences

	Original QI support strategies	Revised QI support strategies	Perceived or reported consequence of revised strategies
Project selection	*Programme approached teams with established plans for projects.* The programme originally approached project teams that had established ideas for projects and had partially developed project plans. The round 1 projects were selected as part of the initial CLAHRC NWL programme funding application process and had not been required to commit to the use of QI methods as a prerequisite.	*Programme invited applications.* Rounds 2 and 3 teams were required to apply to receive support and funding. The application form required use of QI methods, including the model for improvement, and the intended use of QI methods was outlined in guidance documents. Rounds 2 and 3 projects were selected by a peer review process involving academics, clinicians, commissioners and patients. *Preapplication support and QI taster.* Preapplication workshops were run to give potential teams a taster session of the QI methods they would be expected to use within the project.	Project team members cognitively engaged with the subject matter (rather than instantly dismissing or disengaging from use of PDSA method). Teams better understood the expectations on them being part of the CLAHRC NWL programme and receiving funding and support. Taster sessions provided a good foundation to manage expectations.
Teaching style, content and frequency	*Drawing on existing teaching practice.* Teaching materials were taken from other established QI programmes. Two days of predominantly didactic training in QI methods (including PDSA) were provided by external QI experts at the project’s formal start. Training used some interactive elements and frequent use of non-healthcare examples (ie, improving your journey to work) to help understand and practise applying concepts. *Teaching frontloaded in to the project lifetime.* Intensive training provided at the beginning of projects and details of all QI methods described from the outset. Thereafter, 1-day collaborative meetings took place on a quarterly basis (6 in total over 18 months); all project teams came together to receive further light-touch training and participate in peer-to-peer learning about their experiences, including discussion of QI methods. Due to time constraints for recruitment and the need to launch the programme within a given timescale, not all project team members attended the initial 2-day training session. It was intended, however, that the clinical leads and project managers who attended the training would pass on learning to other members of their team as they joined.	*Staggered teaching of methods to times relevant in the project life span.* Project teams were introduced to concepts of why to use QI methods prior to project selection, with basics of the method provided early on and more details of how to use it provided over time as projects progressed. Initial training sessions focused on why to use the method and debates its merits and limitations, and over time evolved to details of conducting a single PDSA well (including critical appraisal of PDSAs from round 1 project teams, and peer-to-peer review of each other’s ‘plan’ stages developed in classroom), before considering iterative chains of PDSA and connection to use of data over time. *More real-life healthcare examples.* Drawing on their first-hand experience of the round 1 projects, examples of using PDSA in healthcare settings were presented and discussed, with the introduction of peer-to-peer learning as round 1 team members joined the teaching faculty. *Debate and critical thinking facilitated.* Teams were encouraged to reflect on their prior experiences of change attempts, and debates were facilitated to explore perceptions on why QI methods might be helpful to address challenges to improvement. Time and space were provided for teams to discuss the pros and cons/good and bad examples of the PDSA cycle method. Teaching material included reflections on other scientific disciplines that used iterative learning approaches (eg, aeronautics, drug development), and interactive debates were built into teaching time to encourage people to share their views and consider why different scientific methods might be suitable for different purposes. Exercises were introduced that promoted critical reflection, such as an interactive game that prompted teams to debate whether to use a PDSA or not in different scenarios. Training sessions encouraged teams to practise applying PDSA to their projects.	Staggered training reduced upfront ‘cognitive load’ and instead provided ‘just in time’ training. Examples were perceived to be of greater relevance and applicability to the new project team members, and less ‘push back’ was experienced. Having past project team members in the room provided credibility for the approach and allowed people to ask questions and explore the reality of what it had been like using PDSA in practice, accessing a depth of ‘real world’ experience. Facilitating debate helped to address the concerns held by some clinical academics about the lack of scientific rigour of PDSA compared with randomised controlled trials and other research methods. Team members could feel that their prior experience and knowledge were heard and valued by the QI support team.
Increased hands on support and QI expertise	*External experts used for training.* External experts provided initial training to internal QI support team and project teams with little ongoing support. The internal QI support team had little or no prior practical experience in PDSA. *Light-touch advisory role.* Support to project teams predominantly offered in an advisory or coaching role before or after using PDSA. *Independent team action.* Project teams used QI methods independently outside of teaching, advisory or coaching sessions. QI support was not invested in to help QI teams use the method.	*Development of QI support team.* Internal QI expertise was developed through working with projects and decreased reliance on external providers. Training and support for internal QI support team members were increased. Time was provided for QI support team members to engage with QI experts (from within and external to the programme) to increase their specialist knowledge about the methods. Opportunity to debate the value of PDSA and to discuss how it should be used in practice was incorporated into internal QI support team meetings. *Hands-on, facilitator support.* QI support team members built relationships with project teams to provide more day-to-day support and active coaching as teams experienced using the method in practice. In round 3 QI support team took greater responsibility to encourage and role-model the use of PDSAs so that project teams could observe PDSA done well and therefore better understand the value of doing so and learn how to do independently. *Wide-ranging support for project teams.* Support to project teams increased to improve scoping of project aims, interventions and development of measure definitions so that data were available to inform PDSA cycles.	QI support staff were able to rehearse ‘difficult conversations’ prior to supporting project teams. QI support staff were prepared to engage in debates in regard to the application of QI methods. QI support staff would encourage teams to use PDSAs by identifying ideas that could be tested through a PDSA, or retrospectively reviewing PDSAs that had already been conducted. QI support staff helped facilitate effective collaborative working within project teams, for example, asking different team members their views on what tests should be conducted or their predictions as to whether change ideas would work or not.

CLAHRC, Collaboration for Leadership in Applied Health Research and Care; NWL, Northwest London; PDSA, Plan-Do-Study-Act; QI, quality improvement.

As the QI support team gained experience and expertise, they recognised that the introduction of PDSA methodology required a fundamental change to how project team members thought about and approached change. This was clearly observed as changes in the reviewed training materials. Interviewees reported that, in light of the observations and the learning gained by the QI support team, deliberate actions were taken to improve the support for PDSA cycle conduct. Table 3 provides details on the original and revised QI strategies, and their reported consequence. Online supplementary appendix 3 provides additional supporting quotes.

10.1136/bmjqs-2017-007605.supp3 Supplementary data

#### Intention to use PDSA

A lack of intention to use the method was reflected in instances of low levels of use and fidelity of PDSA cycle. Qualitative analysis suggested this was influenced by the team’s beliefs and plans on how to tackle change and improvement, as well as their understanding of why the method could be helpful. Some teams had predefined intentions on how to conduct their projects, and clinical academic project team members were also reported to have raised concerns about the iterative nature and small sample sizes that PDSA cycles used.

“to think about changing protocol seemed quite counter-intuitive – [compared to] the more traditional, this is our protocol – we’re going to stick to it – scientific perspective.” (QI support team member, interviewee 2)

There was also a reported perception that the use of PDSA cycles was for the benefit of the QI support team rather than adding value to the project team itself. Areas such as documentation and data collection were seen as a form of programme assurance, rather than as mechanism to help the team learn, and therefore inhibited motivation.

To manage expectations in relation to the use of QI methods, rounds 2 and 3 were required to attend introductory workshops prior to applying for funding and support, and the application process required them to demonstrate their intention to use and initial understanding of QI methods. Changes to training sessions were also made which reflected a recognition that the effective use of PDSA required more than just technical knowledge, but a willingness and motivation to use the method and to change previous ways of working.

“I think the biggest changes we’ve made is trying more to appeal to the hearts and minds of people, so rather than trying to explain it as a technical process was trying to appeal to why might you want to do this? Why might it be useful for your projects and for patients?” (QI support team member, interviewee 1)

Time was also invested for QI support staff to facilitate debate and critical thinking in regard to the method’s use. These discussions were seen as important for project teams to cognitively engage with the PDSA method and position its use within their prior experience and knowledge.

#### Understanding how to use PDSA

Understanding referred to the capability to use the methods and included knowledge of the concept and also the specific principles of the PDSA method. Understanding and intention were distinct factors but interlinked: some teams may have had little intention to use the method as they did not understand it; some may have understood the method and consciously intended not to use it; and some may have intended to use but had insufficient understanding to use with high fidelity.

“It [using PDSA cycles] is still not second nature.” (Project review report—document analysis)

“There were problems with documentation in terms of writing bits of the analysis in the Do section and mixing up the Plan, Do, Study and Act completely.” (QI support team member, interviewee 3)

In some cases, project team members were observed to embrace the PDSA method as an alternative way of working that empowered them to make rapid changes in their local settings. In these cases, however, there was a tendency to ‘PDSA everything’ with little critical consideration of whether the method was being applied well, nor in following the premise of iterative development of a change over time.

As outlined in table 3, original teaching was frontloaded at the beginning of the programme and delivered by external QI experts. Analysis of teaching materials from rounds 2 and 3 demonstrated a different approach that staggered teaching of the method over time, delivered by the QI support team. Initial training sessions focused on the rationale of using the method, and over time evolved to conducting a single PDSA well, before considering iterative chains of PDSA and use of data over time. Training sessions were also designed to include more relevant examples of PDSA cycle use, and individuals with past experience in projects were invited to present them. These examples were perceived to be of greater relevance to the new project team members, and less ‘push back’ was experienced.

#### Application of PDSA in practice

Application of use referred to the way teams went about using the method in practice. It included social challenges, such as bringing a team together to discuss a PDSA, or technical challenges such as the difficulties collecting and analysing data. It was interlinked with the other factors as true understanding could only be achieved through experience of application in practice and the appreciation that the method may be simple in theory but hard to apply in practice.

“The PDSA is in principle a simple tool but in practice it is difficult to use.” (Project review report—document analysis)

Project team members were expected to work together to design, conduct and review PDSAs with the intention of all members sharing their professional perspectives. However, this was reported to rarely occur, and the use of PDSA was often delegated to an individual team member. Practical time constraints or competing priorities also presented challenges to completing PDSA in real time and with high fidelity. The method was often used retrospectively to frame past actions rather than prospectively plan and test changes iteratively. This meant that principles such as use of predictions or consideration of scale were not applied.

“I don’t know if there would be many teams who would use it in a daily project meeting – sit around and say well this was the PDSA we said we were going to do – how did it work out? I think it was still a bit more of one person’s responsibility.” (QI support team member, interviewee 1)

“PDSA are currently being written up retrospectively rather than as the test is happening.” (Project team report)

In recognition of these challenges, the role of QI support team shifted from an arm’s length advisory role to working much more closely with the project teams. This included greater presence of QI support staff within projects to facilitate structured discussion about how changes should be tested and role-model the use of PDSAs. Teams were also supported to develop aims and measure definitions earlier so that timely data were available to inform PDSA cycles.^18^ Additionally, the programme invested in providing greater support and training to the QI support team members themselves.

“We didn’t want them to rush off and change practice, we wanted them to sort their measures out and get their baselines and then test changes.” (Interviewee 2)

## Discussion

Over a three-and-a-half-year study period, moderate yet significant improvements were seen in the number of PDSA cycles conducted and the fidelity of these cycles against the key principles of the method. However, across the total sample of projects, PDSA cycle fidelity remained low with key principles of the method frequently not met. The study presents a theoretical framing and practical solutions to support better use of the PDSA method. It suggest that project teams’ intention, understanding and application of the PDSA cycle method are three areas in which QI support teams should consider when supporting the method’s use.

The study reiterates previous findings suggesting that the PDSA cycle methods,^3 6^ and QI methods in general,^19 20^ are not always applied as they are intended. The study also provides a detailed reflection on how QI methods are introduced influences their uptake and use, adding to the literature on the influence of context on QI approaches.^21–24^ This provides empirical grounding to support recent claims cautioning against the oversimplification of QI methods when they are taught and adopted into mainstream healthcare practices, demonstrating that the full benefits of these methods are often not realised^25 26^ and suggesting that challenges can be accentuated when the use of QI methods is new to individuals or teams.^27^

The actions taken by the QI support team to revise the QI support strategies align with improvements in fidelity of PDSA cycles. Given some limitations of the retrospective nature of this work, findings about the relationship between changes in QI support strategies and improvements in PDSA fidelity are intended to be exploratory (hypothesis-generating) rather than indicative of causality (hypothesis-confirming). The programmed yearly project initiation cycle allowed an iterative approach to be taken by the QI support team to respond to challenges faced and revise QI support strategies over time. The QI support strategies were revised in the recognition that developing intention to use PDSA, understanding of how to use it and mastery of its application in practice are a gradual and negotiated process.

Of note, the measures of fidelity that did not see significant improvements were those requiring users to revisit the method, including the increasing scale and use of data over time. Even with an adequate level of intention and understanding, these principles are arguably more complex and harder to achieve as they require skills and behaviours to work effectively as a multidisciplinary team to make decisions and plan between cycles, as well as the application of ‘measurement for improvement’ principles.

In establishing a rationale for the changes in fidelity over time, we also considered other changing contextual factors. Over the study period, the majority of the QI support team stayed with the programme and were likely to have gained in skills and competencies over this time. A small number of project team members also moved between teams over rounds. This could provide an alternative explanation as individuals increase experience and understanding of how to use PDSA over time, although perceptions from the QI support staff indicated that disengagement and misunderstanding of the method were equally likely to persist over rounds for some individuals. The authors are not aware of any other major contextual influences that happened during the study period. The extent of these or other contextual influences remains to be tested in future research.

### Implications

This study reinforces growing research that emphasises that the use of QI methods is not simple. The use of QI methods must be considered as complex sociocultural interventions that require significant technical and social skills. This understanding needs to inform future use and the design of QI support strategies and PDSA education.

Through the challenges of intention to use and understanding and application of the methods in practice, this research raises questions about the most appropriate teaching, training and support mechanisms required for effective use of QI methods. The findings present new learning to inform the design, delivery and evaluation of QI training including PDSA cycles. Frequently QI training is perceived as deliverable in a short period of time^28 29^; however, existing studies have demonstrated the limited impact of such approaches in the rigorous and effective use of QI methods.^27 30^

Previous studies have identified the challenges of adopting new methods into existing organisational cultures and practices,^19 20 31–33^ and that to use QI methods well requires people to adopt fundamentally different ways of working.^31 32^ Introducing QI approaches into new settings needs to be carefully designed and delivered to consider how to support the introduction of behaviours that are often counter to prevailing organisational norms. The idea of conducting pragmatic and scientific tests of change locally to ensure that interventions were fit for purpose in a particular setting occupies a middle ground between the rigour of traditional academic research and the pace of change in healthcare organisations. While PDSA has the potential to bridge between these two worlds, it also presents a very different way of working which was not readily accepted or implemented by teams using the method.

### Future work and limitations

This is the first study to provide a detailed assessment of a large number and range of PDSA cycles documented in real time during an improvement project. The fidelity assessment framework presented in this paper could be used in the future to provide a formative assessment of PDSA fidelity and provision of real-time feedback to project teams. This can support teams to identify and respond to factors within their local context and improve PDSA cycle conduct.

While this research would ideally have been conducted prospectively, the challenge of PDSA use and how to improve it only drew the attention of programme leaders, and researchers, as the work progressed.^34^ A resulting limitation is that interviews were only conducted with the three QI support staff who had been present and involved in teaching across the different project rounds and were still working with the programme at the time of the study. To counter this challenge, common themes were drawn from triangulating data from project team reports and review meeting minutes, training material and QI support staff interviews to ensure findings were reflective of the project team’s perspectives as well as the QI support team. The theories and strategies proposed in this paper require further investigation and, in particular, prospective application to assess if improvements in fidelity can reliably be achieved in practice.

Additionally, a limitation is that the study was reliant of PDSA cycles documented by front-line teams. While this provides a greater depth of insight to published reports of PDSA,^3^ it provides only a partial and selective reflection of how PDSAs were used in practice. Further research is needed to observe actual PDSA conduct in practice and to understand the perspective of front-line QI teams.

A further limitation of the study is that PDSA cycles were only assessed quantitatively against the principles of use, with no qualitative assessment of the nature of changes made, nor the success of the learning and adaptions introduce through subsequent cycles. The principles can therefore be considered necessary, but not sufficient, to determine the quality of PDSA cycle use. As such, this study simply reports on a minimum standard of PDSA fidelity. The findings demonstrate that engaging and motivating people to use PDSA at all and achieving these minimum standards in themselves are challenging, and therefore provide learning to others. Future research would, however, benefit from including additional work to understand how change ideas are adapted, with and without success, over time.^7^

## Conclusion

This study demonstrates that PDSA fidelity can improve over time and identifies revisions made to QI support strategies intended to influence the intention and motivation of project teams to use PDSA, and their understanding and application of the method in practice. The study reinforces the literature that suggests engagement and fidelity in using QI methods are challenging, and that QI methods should be considered as complex sociocultural interventions that also require significant technical skill. The work suggests that QI support strategies can be designed to support increased PDSA use and fidelity, but that achieving this is a gradual and negotiated process requiring sufficient time and support to explore different perspectives and encourage new ways of working.
