# Supplementary material for: Evolving quality improvement support strategies to improve Plan–Do–Study–Act cycle fidelity: a retrospective mixed-methods study
Source: BMJ Qual Saf. 2019 Mar 18;28(5):356–65. doi: 10.1136/bmjqs-2017-007605 (PMC6560463; doi:10.1136/bmjqs-2017-007605)
Supplement: Supplementary data [file bmjqs-2017-007605supp001.pdf]

Appendix 1 – Supplementary method and results

Methods

How did PDSA cycle fidelity change over time?

The quantitative outputs for the measures of fidelity from the first stage of analysis were divided by the year the project teams were initiated. A one-way ANOVA and post-hoc t-tests were firstly used to determine change in the mean number of PDSA cycles conducted per project overtime.

Secondly, each variable was arranged in a dichotomous manner in relation to fidelity; whether the principle was adhered to or not. Using a Chi-square test and a subsequent trend test, the Marascuilo procedure, the observed adherence to each measure was compared to the expected adherence based on the proportion of projects teams initiated in each year.[15]

Chi-Square

Pearson's Chi-square test for independence evaluates how likely it is that any observed differences between categorical data sets arose by chance.[15] It is used in this case to determine change in fidelity between PDSA cycles documented by improvement teams of the three rounds.

Chi-square tests the null hypothesis and indicates whether it can be rejected or not. If it is rejected it indicates that observed differences between the fidelity of PDSA cycles across Rounds is not due to chance. In this study the Null hypothesis, across any principle reflecting an aspect of PDSA cycle conduct fidelity, was that the fidelity of PDSA cycle principles is the same across each Round of projects.

Observed data for each principle was compared to the expected distribution. In this case, for the PDSA cycle principles reviewed, Chi-square tested the observed distribution figures across Rounds of projects against the expected distribution as indicated by the number of PDSA cycles based on the sample and the distribution of PDSA cycles between each Round.

Marascuillo procedure

Whilst rejecting the null hypothesis using Chi-square indicates that the differences between Rounds are unlikely to be due to chance, it does not indicate between which Rounds there are the significant differences. The Marascuilo procedure, a multiple comparisons test, was used to determine these trends.[15] The Marascuilo procedure allows comparisons between all pairs of groups. If the null hypothesis is rejected for a variable the procedure was used to determine between which Round any difference is significant.

Role of researchers

CM and LL collected data, interviewed participants and carried out the analysis. Whilst employed by NIHR CLAHRC NWL, they joined later in the programme and had no involvement in supporting or provision of training to project teams. TW supported PDSA cycle data extraction and quantitative analyses. JR and DB provided academic oversight and support to the study and review of analysis. JR and DB were involved in development of the QI support strategies, teaching of PDSA and overall programme oversight but not day to day project support. All researchers contributed to reflections and sense making following initial analysis of results by LL and CM and contributed to the development of the article manuscript.

Results

What was the fidelity of conduct of all PDSA cycles against core principles of the method?

A one-way ANOVA indicated that the null hypothesis that all means are equal across Rounds can be rejected and that the differences were not due to chance ($F(2,36)=4.64$, $p<.05$) (Appendix table 1).

Groups	Number of projects	Number of PDSA cycles	Mean number of cycles per project	Variance
**Round 1**	6	30	5	34
**Round 2**	16	144	9	27.3
**Round 3**	17	247	14.5	82.6

Source of Variation	SS	df	MS	F	P-value
**Between Groups**	490.1	2	245.1	4.6	0.016
**Within Groups**	1902.2	36	52.8		
**Total**	2392.4	38			

SS, sum of squares; df, degrees of freedom; MS, mean square; F denotes the F statistic used with Anovas

APPENDIX TABLE 1. PDSA CYCLES INITIATED BY CLAHRC NWL PROJECT TEAMS
