# Supplementary material for: Evolving quality improvement support strategies to improve Plan–Do–Study–Act cycle fidelity: a retrospective mixed-methods study
Source: BMJ Qual Saf. 2019 Mar 18;28(5):356–65. doi: 10.1136/bmjqs-2017-007605 (PMC6560463; doi:10.1136/bmjqs-2017-007605)
Supplement: Supplementary data [file bmjqs-2017-007605supp002.pdf]

Appendix 2 – Full statistical analysis of change in measures of PDSA cycle fidelity over round of project initiation

		Observed data						*Chi-Square test*				*Marascuilo Procedure*			
Principle	Measure		*Round 1*	*Round 2*	*Round 3*	*All*	*Line Chart*	*DoF*	*Sample size*	*Chi-Square value*	*Significance level, p*	*Contrast: From Rx to Ry*	*Value*	*Critical range*	*Significant*
Documentation	All PDSA cycle stages documented	Cycles adhering to principle	15	93	191	299	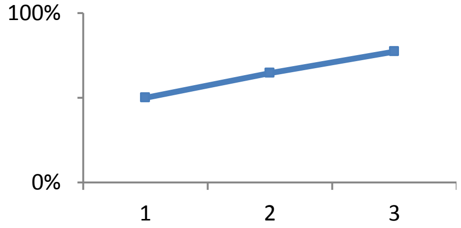					R1-R2	0.15	0.24	No
		Cycle Sample	30	144	247	421		2	421	14.11	< 0.001	R1-R3	0.27	0.23	Yes
		%	50.0%	64.6%	77.3%	71.0%						R2-R3	0.13	0.12	Yes
	"Study" documented in past tense	Cycles adhering to principle	10	67	176	253	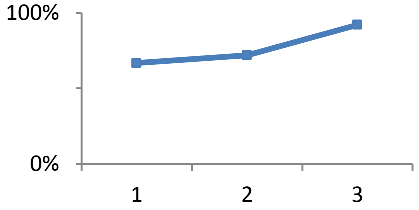					R1-R2	0.05	0.32	No
		Cycle Sample	15	93	191	299		2	299	23.33	< 0.001	R1-R3	0.26	0.3	No
		%	66.7%	72.0%	92.1%	84.6%						R2-R3	0.2	0.12	Yes
Learning Activity	Learning Activity present	Cycles adhering to principle	15	90	189	294	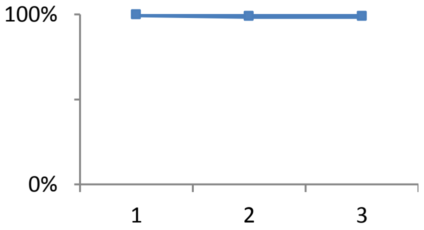								
		Cycle Sample	15	93	191	299		N.A*				N.A*			
		%	100%	96.8%	99.0%	98.3%									
Prediction	Explicit Prediction documented	Cycles adhering to principle	0	3	33	36	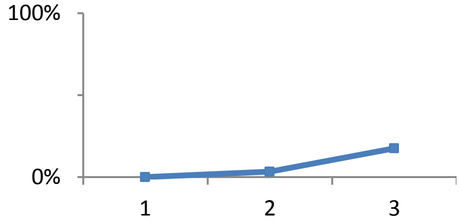					R1-R2	0.03	0.05	No
		Cycle Sample	15	90	189	294		2	294	13.53	0.001	R1-R3	0.18	0.07	Yes
		%	0.0%	3.3%	17.5%	12.2%						R2-R3	0.14	0.08	Yes

Iterative Cycles	PDSA cycle within iterative series of cycles	Cycles adhering to principle	0	48	115	163	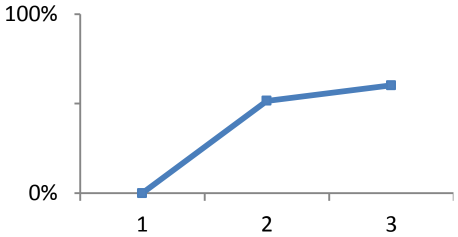					R1-R2	0.52	0.13	Yes
		Cycle Sample	15	93	191	299		2	299	20.79	< 0.001	R1-R3	0.6	0.09	Yes
		%	0.0%	51.6%	60.2%	54.5%						R2-R3	0.09	0.15	No
Small scale testing	PDSA iterative series increasing testing scale	Iterative series adhering to principle	N.A	3	16	19	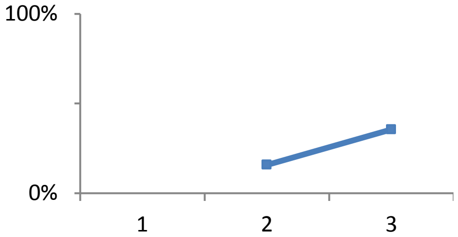								
		Cycle Sample	N.A	19	45	64		1	64	2.5	0.113				
		%	N.A	15.8%	35.6%	29.7%									
Use of Data overtime	PDSA iterative series using regular data over time	Iterative series adhering to principle	N.A	7	22	29	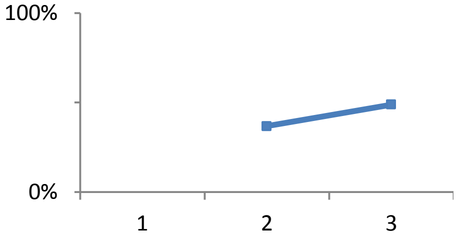								
		Cycle Sample	N.A	19	45	64		1	64	0.782	0.376				
		%	N.A	36.8%	48.9%	45.3%									

Appendix table 2. Change in measures of PDSA Cycle fidelity over Round of project initiation (The table depicts three areas of results: 1) The observed data over all and each individual Round, the total cycle sample and a percentage. 2) Results from Chi-Square tests. 3) Results from Marascuillo procedure)

*As a predeteminant to running the test, Chi square test requires 80% of the expected frequencies to exceed 5 data items, in this case 5 PDSA cycles. This was the case for all categories in question except "Learning activity presence". Frequencies were high across all Rounds and therefore the expected frequency of not having a learning activity present did not exceed 5 PDSA cycles.
**Increasing scale of testing and use of data over time categories were dependent on iterative cycles. As no iterative series were present in Round 1 comparisons within the categories were made using Chi-square for only Round 2 and 3.
