# Supplementary material for: Evolving quality improvement support strategies to improve Plan–Do–Study–Act cycle fidelity: a retrospective mixed-methods study
Source: BMJ Qual Saf. 2019 Mar 18;28(5):356–65. doi: 10.1136/bmjqs-2017-007605 (PMC6560463; doi:10.1136/bmjqs-2017-007605)
Supplement: Supplementary data [file bmjqs-2017-007605supp003.pdf]

Appendix 3 – Supporting quotes for revised QI strategies

	Supporting quotes
Project selection	*"the pre-application stuff, the workshop, was a conscious effort to make it clearer to projects what we expected in terms of usage of methodology because we had struggled so much" (QI support team member)*<br><br>"[We were] actively structuring the application form around the tools and techniques to give [the project teams] a head-start" *(QI support team member)*
Teaching style, content and frequency	*"We'd got further towards what we would describe as good practice in teaching in terms of having good examples, relevant examples and peer to peer input" (QI support team member)*<br><br>*"The opportunity to speak to and learn from others is highly valued by the [project] team members, as well as the use of real examples in the process mapping and PDSA sessions" (Project team report)*<br><br>*"using examples from other people in the peer group, both good and bad in order for participants to have some form of basis for critical reflection which they could then take in to their own practice" (QI support team member)*<br><br>*"At the end of the session you will… have practice-based experience of developing and undertaking PDSA cycles in generic and healthcare-based scenarios" (Training material)*
Increased hands on support and QI expertise	*"[QI support team member] suggested doing this as a PDSA to test whether it affects follow up rates" (Project team report)*<br><br>*"[the deployed support staff] had the opportunity to do some of the more basic things like documenting PDSAs during meetings." (QI support team member)*<br><br>*"there have been instances of more dedicated roles, where people have played more of a hybrid role as part QI support team person but part project person and I think those roles have really helped people to see PDSAs as being used well in practice" (QI support team member)*<br><br>*"I remember feeling in round two that we had to keep an eye on the training in our own team as well because I think with quite a lot of new people who hadn't seen [the external] original training, I remember thinking at the time there were people in our own team who didn't get some of the same issues" (QI support team member)*<br><br>*"think having the written criteria and going through some examples then helped in our own team to build a capability to train in this stuff" (QI support team member)*

APPENDIX TABLE 3. SUPPORTING QUOTES FOR REVISED QI STRATEGIES
